# The 6th Iberian and 2nd Ibero-American Cyanotoxin Congress CIC2019

**DOI:** 10.3390/toxins13020162

**Published:** 2021-02-19

**Authors:** Marina Aboal

**Affiliations:** Laboratory of Algology, Plant Biology Department, Faculty of Biology, Murcia University, 30100 Murcia, Spain; maboal@um.es

According to genomic data, toxin cyanobacteria production is likely as old as the group itself [1], and environmental and associated health problems could be tracked to 1000 years ago in China [2], Scotland lochs in the twelfth century [3], or in Poland in the seventeenth century [4]. However, the worldwide toxicity events caused by cyanobacteria are a contemporary issue that is becoming a real threat to the environment and the human population.

From the very beginning, most research efforts have globally focused on studying planktonic species due to sanitary implications [2], and also given the fact that they also soon detected toxic benthic species causing cattle and pets fatalities [5,6], and afterwards detected toxic mats with a high frequency [7,8,9].

First, the environmental and sanitary problems associated with cyanobacteria proliferations were detected in Northern and Central Europe, but similar situations were rapidly described in South Europe. It was in this context that the researchers involved in algae toxicological problems and water resources management decided to create a Spanish Cyanotoxins Network to ease communication and collaboration between groups for the sake of common methodologies, and to be ready to offer efficient answers to potential problems. The network quickly grew with the incorporation of Portugal to become Iberian. Then, periodic meetings were enriched by the presence of Ibero-American colleagues.

The meetings held every 2–3 years mirrored problems of the time and showed an astonishing diversity in the developed issues, of which volume 34 of Limnetica (2015), and 9 of *Toxins* (2017), are good examples of the heterogeneity of contributions. The last venue (Murcia, SE Spain) welcomed researchers from three continents and eight countries, who presented papers that focused on fairly different aspects, ranging from tele-detection to the identification of infrequently reported toxins, as well as the quantification of beneficial and harmful molecules.

The scientific production related to cyanotoxins in countries like Portugal has been enormous in recent years. Moreira et al. [10] presented an update of the current situation by focusing on the recent increase in both toxin events and the number of detected toxic compounds, and the importance of the methodologies followed.

Some fairly rare toxins may abound locally and may represent important hazards that affect people, animals, and even crops. Anabaenopeptins (A, B, C, and F) and Oscilamide Y were detected at unusually high concentrations in a reservoir system in NE Spain [11] and [D-Leu^1^]MC-LR is more frequent than MC-LR in some South American countries [12]. The detection of some of these compounds may pose a challenge, and Flores and Caixach [11] recommend taking a suspect screening approach based on High-Resolution Mass Spectrometry (HRMS) for the cyanobacterial peptides that are not often monitored by target strategies. [D-Leu1] MC-LR is frequently reported in water bodies throughout America and shows clear toxic potency differences with MC-LR, with greater phosphatase activity inhibition in both animal and plant models. The effects on plants (*Phaseolus vulgaris*) can be observed from germination at fairly low concentrations [12,13]. This may be a warning for not only managers to be aware of most minority compounds that challenge the accuracy of methods, but also for researchers to study the potential toxic effects of Anabaenopeptins and Oscilamide, among others.

Besides the large volume of publications on the subject, we still do not know the factors that trigger cyanobacteria toxicity and we are still likely far from understanding (and preventing) toxic events than knowing the parameters that promote cyanobacteria blooming. Very early observations showed the influence of cyanotoxin production on the diversity of aquatic communities, at least in calcareous rivers [14,15], but almost nothing has been done on this subject since then.

Some cyanobacteria (*Spirulina/Arthrospira*) have been, and are presently, important sources of proteins for an increasing proportion of people, and have been proposed as an alternative source of proteins in developing countries to fight against malnutrition [16]. However, the presence of microcystins has been previously reported and is confirmed in the food supplements containing them [17] at concentrations below the level of quantification. The presence of microcystins and anatoxin has been detected in a wide variety of habitats [9], but almost always at very low concentrations.

Cyanobacteria are not the only consumed microalgae as *Chlorella* and other green algae can be obtained everywhere. However, a large body of knowledge has accumulated about the toxicity of some commercial microalgae products [18], especially those collected directly from nature. In these supplements, high levels of heavy metals and other potentially harmful compounds have also been detected [18]. Adult populations are probably safe because limits are not usually exceeded, but it is not the same with children, as recommended intakes are not normally indicated, and children are the most fragile part of our population, especially when daily intake is recommended. Much better control should be applied to the labeling of these products to always indicate not only the origin of cultures or collections, but also production and preservation methods, and a toxicological analysis, which should be compulsory as it is in other foods.

Methodological advances are a crucial part of cyanotoxins research. It is very important to permanently continue to search for the most accurate, inexpensive, and quickest way to identify and quantify the increasing number of cyanotoxins in both continental and marine environments [17].

The most fascinating characteristic of cyanobacteria is probably their capacity to produce not only a vast number of harmful compounds, but also high concentrations of antioxidants, cytoprotectors, pigments, and other biotechnologically interesting chemicals [19]. The biochemistry of these organisms remains quite unknown, but several new potentially useful molecules have been recently identified and named [20], and the list of beneficial activities continues to grow [21].
